# Efficacy of “Dihuang pill prescriptions” combined with conventional treatment for diabetic kidney disease: A network meta-analysis and systematic review

**DOI:** 10.1097/MD.0000000000035290

**Published:** 2023-09-29

**Authors:** Minghao Lin, Hui Zhang, Shilin Liu, Andong Li, Zheng Nan

**Affiliations:** a School of Traditional Chinese Medicine, Changchun University of Traditional Chinese Medicine, Changchun, China; b Department of Endocrinology and Metabolism, The Affiliated Hospital to Changchun University of Chinese Medicine, Changchun, China.

**Keywords:** diabetic kidney disease, Jingui Shenqi Pills, Jisheng Shenqi Pills, Liuwei Dihuang Pills, Meta-analysis, Zhibai Dihuang Pills

## Abstract

**Background:**

With the increasing incidence of diabetic nephropathy, there is currently no means to completely cure the disease. However, a large number of clinical data proved that traditional Chinese medicine combined with modern medical conventional treatment of diabetic kidney disease has achieved better efficacy than simple Western medicine conventional treatment.

**Methods:**

Based on the mesh meta-analysis method, the objective evaluation of clinical efficacy of conventional treatment of diabetic kidney disease and comparison provided more evidence-based basis for the treatment of diabetic kidney disease and further select effective intervention measures to delay the process of diabetic kidney disease.

**Results:**

41 randomized controlled trials were included, involving 4 kinds of “Dihuang pill prescriptions,” with a total sample size of 3562 cases, including 1793 patients in the experimental group and 1769 patients in the control group. Network meta-analysis suggested that the best SUCRA-ranked 2 interventions were Jingui Shenqi pills/decoction + Western medicine routine” and Jisheng Shenqi pills/decoction + Western medicine routine in terms of reducing 24-hour urinary protein. In terms of reducing urinary albumin excretion rate, the top 2 SUCRA-ranked interventions were Zhibai Dihuang pills/decoction + Western medicine routine and Liuwei Dihuang Pills/decoction + Western medicine routine. In terms of reducing serum creatinine, the top 2 SUCRA ranked interventions were Jisheng Shenqi pills/decoction + Western medicine routine, Zhibai Dihuang Pills/decoction + Western medicine routine. In terms of lowering fasting blood glucose, the top 2 SUCRA-ranked interventions were Zhibai Dihuang pills/ decoction + Western medicine routine and Jisheng Shenqi pills/decoction + Western medicine routine. The results showed that the treatment plan of conventional Western medicine combined with Chinese patent medicine could reduce serum creatinine, 24-hour urinary protein, fasting blood glucose urine protein excretion rate and improve the total clinical effective rate.

**Conclusion:**

The combination of medicine was obviously better than conventional Western medicine alone.

## 1. Introduction

From 2000 to 2015, the number of patients with end-stage renal disease complicated with diabetes increased from 19.0% to 29.7%, and the number of patients with end-stage renal disease caused by diabetes increased from 22.1% to 31.3% during this period.^[1]^ According to the statistics analysis of the International DM Alliance, there will be 693 million diabetes patients in 2045.^[2]^ Molitch ME pointed out that without special intervention, 75% of patients with diabetes will develop end-stage renal disease within 20 years.^[3]^ At present, there is no special method to cure diabetic kidney disease. Modern medicine mainly focuses on symptomatic treatment such as lowering blood sugar, lowering blood pressure, regulating blood lipids and lowering urine protein. Although its curative effect is considerable, it still has its limitations. In recent years, the curative effect of Chinese traditional medicine in the treatment of diabetic kidney disease is relatively considerable. A large number of clinical studies have found that conventional treatment of diabetic kidney disease with traditional Chinese medicine combined with Western medicine can significantly reduce urinary protein excretion, reduce 24-hour urinary protein, reduce Serum creatinine level, and delay the progression of diabetic kidney disease. In this paper, 4 types of literature on “Dihuang pill prescription” combined with conventional treatment will be extracted for strict inclusion analysis, and then the basic information of the literature will be included and the quality of the literature will be evaluated. Stata 17.0 software will be used to analyze the comparative relationship diagram between the interventions, and finally, the results will be analyzed and the shortcomings of improvement strategies will be made.

At present, the preparation of “Dihuang Pills prescription” is widely used in the treatment of diabetic kidney disease. Such as the composition of Liuwei Dihuang Pills living ground, Danpi, gardenia, yam, dogwood, poria. Jingui Shenqi Pills is based on Liuwei Dihuang Pills with cassia branch and aconite. Zhibai Dihuang Pills is in Liuwei Dihuang Pills on the basis of adding common anemarrhena, phellodendron. At present, “Dihuang Pills prescription” to pills or decoction is widely used in clinics. Therefore, based on the network meta-analysis method, this study objectively evaluated and compared the clinical efficacy of “Dihuang Pills prescription” combined with conventional Western medicine in the treatment of diabetic kidney disease, so as to provide more evidence-based evidence for the treatment of diabetic kidney disease with traditional Chinese medicine preparations, and further select effective intervention measures to delay the course of diabetic kidney disease.

## 2. Data and methods

### 2.1. Literature search

Chinese and English databases such as CNKI, Pillsfang, CBM, EMbase, Web of Science, Vip, PubMed and Cochrane Library were searched by computer. Chinese database search terms include “diabetic nephropathy,” “diabetic kidney disease,” “Xiaoke nephropathy,” “diabetic nephropathy,” “Liuwei Dihuang Pills,” “Shenqi Pills,” “Jisheng Shenqi Pills,” “Zhibai Dihuang Pills,” “Chinese patent medicine,” etc. The keywords in English are diabetic nephropathy, diabetic kidney disease and diabetic nephropathy. The search terms are connected with or and/or and respectively to form search expressions. The randomized controlled trials included in the database up to April 2023 were screened using a combination of subject terms and free words.

### 2.2. Inclusion criteria of literature

#### 2.2.1. Research types.

It is a clinical RCT of Dihuang pills mainly used in the treatment of DKD. No matter whether blind method is adopted or not, the published language is limited to Chinese and English, and the region is not limited.

#### 2.2.2. Research objects.

Patients with DKD diagnosis. The diagnostic criteria are in line with a 2014 agreement between the American Diabetes Association and the Kidney Foundation.

#### 2.2.3. Intervention measures.

The control group was treated with conventional DKD therapy, including the general treatment of diabetic nephropathy (nutrition, lifestyle, weight control), blood glucose control, blood pressure control, lipid regulation (no limitation of dosage forms, dosages, and types of drugs). On the basis of the control group, the experimental group was added with Dihuang pills or traditional Chinese medicine compound with Dihuang pills as the main drugs (no limitation of dosage forms and dosages, including decoction, pill, powder, paste, granule, etc.) to treat diabetic nephropathy.

#### 2.2.4. Outcome indicators.

Included outcome indicators: Effectiveness evaluation indicators: serum creatinine, urinary protein excretion rate, 24-hour urinary protein, fasting blood glucose, adverse reactions, good uniformity between groups and comparable research data.

### 2.3. Exclusion criteria

Literatures that do not meet the above inclusion criteria; Repeated publication, incomplete or incorrect data, improper use of statistical methods; non-randomized controlled trials; In addition to “Dihuang Pills prescription” and conventional treatment, the treatment group took other traditional Chinese therapy, such as acupuncture, massage, plaster, etc.; Included patients with other diseases affecting renal function, such as serious cardiovascular and cerebrovascular diseases, lung diseases, liver diseases, nephrotic syndrome, urinary tract infection, chronic glomerulonephritis, hypertensive nephropathy and other literatures affecting urinary protein were deleted.

### 2.4. Literature screening and data extraction

The collected literatures were imported into the Endnote literature management software to check for duplications and then removed. Two researchers (Minghao Lin, Hui Zhang) extracted the literatures according to the detected literatures, and conducted independent screening and evaluation according to the inclusion criteria and exclusion criteria. If there is any difference of opinion, Join another researcher (Shilin Liu) to discuss opinions or make a final assessment. Basic data of the included literature were extracted, including the first author of the literature, publication year, sample size, outcome indicators, age, course of treatment, adverse reactions, etc. In the extraction process, it is necessary to verify the original index of the literature. If there is fuzzy information or errors, the original author will be contacted through the email address left in the literature. If the authenticity of the original data cannot be proved, whether to abandon it will be considered and the quality of the included literature will be strictly controlled.

## 3. Result

This article is registered with Prospiro (registration number: CRD42023416484).

### 3.1. Literature retrieval

**Figure 1.**
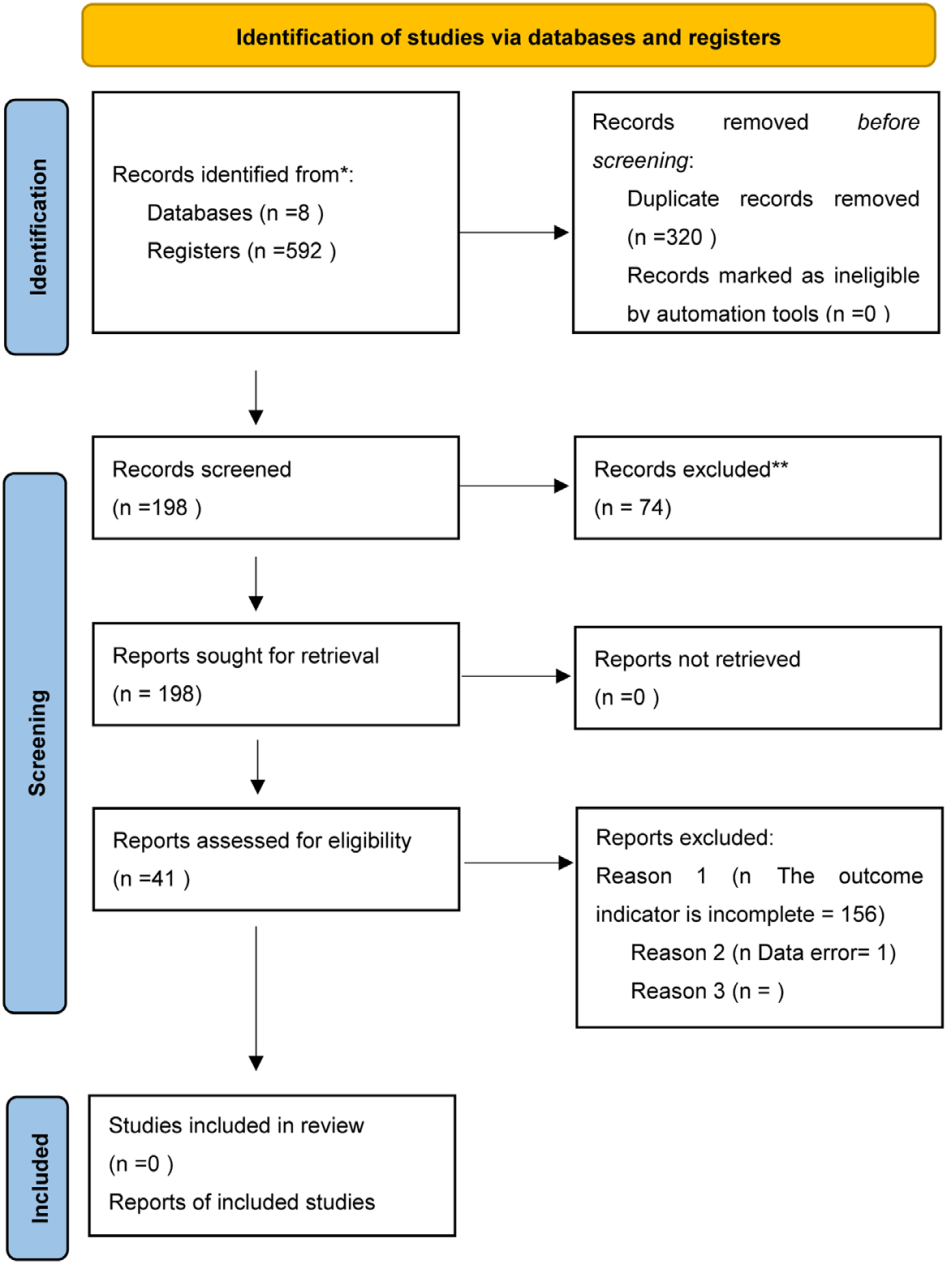
Data extraction table.

A total of 592 literatures were retrieved, and a total of 41 literatures were included after layer by layer screening, with a total of 3562 patients, including 1793 patients in the experimental group and 1769 patients in the control group. The literature retrieval process is shown in the Figure 1.

### 3.2. Basic information of the included literature

A total of 4 preparations of “Dihuang Pills” have been included, including Liuwei Dihuang Pills, Jingui Shenqi Pills, Jisheng Shenqi Pills and Zhibai Dihuang Pills. The basic characteristics of the included literatures are shown in Table 1.

**Table 1:** The basic characteristics of the included literatures.

Document source	Sample size	Male/female/case		Average age		Average course of disease					
	T/C	T	C	T	C	T	C	Intervention	Course of treatment per wk	Outcome index	Adverse reaction
Li Dongmei 2023^[4]^	49/49	27/22	26/23	58.60 ± 5.02	58.65 ± 4.78	7.60 ± 2.53	7.62 ± 2.56	LW + CT	8	③	Report
Ma Yaping 2015^[5]^	74/74	39/35	30/44	57.00 ± 5.80	54.00 ± 5.80	5.70 ± 1.50	5.20 ± 1.70	LW + CT	36	②	Report
Liu Chaoju 2012^[6]^	30/30	17/13	16/14	63.75 ± 5.85	62.75 ± 5.75	–	–	LW + CT	8	③	Unreported
Li Xuexia 2011^[7]^	30/30	18/12	19/11	52.73 ± 12.61	52.98 ± 13.46	8.92 ± 3.63	7.29 ± 4.21	LW + CT	12	①	Unreported
Xie Jiajia 2011^[8]^	30/30	17/13	16/14	50.70 ± 5.32	51.20 ± 6.13	12.30 ± 2.80	12.10 ± 3.50	LW + CT	12	②④	Unreported
Zhou Junping 2021^[9]^	34/34	18/16	19/15	67.80 ± 3.50	67.24 ± 3.15	7.15 ± 1.12	7.20 ± 1.15	LW + CT	4	③④	Report
Feng Sanhua 2015^[10]^	60/60	35/25	33/27	46.30 ± 3.80	46.70 ± 4.00	8.30 ± 1.50	8.40 ± 1.70	LW + CT	12	①④	Unreported
Li Yun 2019^[11]^	52/52	31/21	30/22	58.94 ± 8.75	59.23 ± 8.84	8.61 ± 5.29	8.52 ± 5.33	LW + CT	8	①③④	Unreported
Jia Fan 2017^[12]^	65/65	33/32	35/30	52.47 ± 2.85	52.07 ± 2.73	–	–	JG + CT	–	②③④	Unreported
Hou Cuirong 2017^[13]^	45/45	24/21	23/22	61.38 ± 10.36	61.84 ± 10.46	8.31 ± 2.16	8.62 ± 2.32	JG + CT	–	②③④	Unreported
Sun Hongyun 2018^[14]^	33/31	18/15	17/14	55.60 ± 11.20	54.80 ± 11.40	–	–	JG + CT	–	②③④	Unreported
Jiang Bin 2020^[15]^	60/60	35/25	34/26	54.65 ± 5.50	55.20 ± 5.24	3.26 ± 1.43	3.50 ± 1.51	JG + CT	4	②④	Unreported
Zhang Jingzu 2019^[16]^	118/118	68/50	67/51	53.10 ± 3.10	53.70 ± 3.10	3.20 ± 0.30	3.10 ± 0.30	JG + CT	4	②④	Unreported
Li Caijing 2018^[17]^	40/40	24/16	23/17	66.30 ± 6.20	66.20 ± 6.30	6.90 ± 1.20	6.80 ± 1.30	JG + CT	4	①②④	Unreported
Wang Zheng 2019^[18]^	43/41	29/14	28/13	57.60 ± 7.90	57.20 ± 8.10	9.50 ± 2.20	9.10 ± 2.40	JG + CT	12	②	Report
He Jianjun 2016^[19]^	39/39	24/15	22/17	55.00 ± 4.80	55.10 ± 5.00	3.40 ± 0.90	3.20 ± 0.70	JG + CT	4	①②④	Report
Liu Dongmei 2022^[20]^	62/62	31/31	32/30	54.03 ± 3.39	53.96 ± 3.28	–	–	JG + CT	4	②③④	Unreported
Tan Jian 2014^[21]^	22/21	12/10	11/10	59.72士3.17	61.32 ± 2.27	–	–	LW + CT	8	②③④	Unreported
Sun Guisheng 2012^[22]^	35/35	20/15	21/14	57.33 ± 11.54	58.94 ± 11.36	3.35 ± 1.23	3.14 ± 1.05	JG + CT	4	②③	Unreported
Zhang Hongxia 2009^[23]^	35/33	18/17	17/16	61.98 ± 5.69	62.06 ± 5.52	7.93 ± 1.65	7.96 ± 0.96	JS + CT	4	①②③	Unreported
Deng Cui 2019^[24]^	34/34	22/12	21/13	–	–	7.66 ± 2.39	7.43 ± 2.46	JS + CT	8	①②④	Unreported
Xiong Manqi 2003^[25]^	40/38	23/17	20/18	46.35 ± 4.17	43.58 ± 4.16	4.32 ± 4.21	4.27 ± 2.15	JS + CT	6	②③④	Unreported
Luo Yifeng 2021^[26]^	25/25	14/11	13/12	49.22 ± 3.17	49.18 ± 3.26	–	–	JS + CT	8	②④	Unreported
Li Minji 2018^[27]^	43/43	24/19	25/18	47.24 ± 9.45	48.62 ± 9.78	5.87 ± 3.87	5.27 ± 4.14	JS + CT	8	①	Unreported
Li Shuang 2020^[28]^	50/50	26/24	27/23	55.10 ± 9.40	54.60 ± 8.90	10.20 ± 1.10	9.80 ± 0.90	JS + CT	8	③④	Unreported
Wang Yongzheng 2015^[29]^	30/30	14/16	12/18	59.10 ± 9.10	53.50 ± 9.50	–	–	LW + CT	–	①④	Unreported
Xie Long 2019^[30]^	78/77	45/33	44/33	61.47 ± 8.46	61.46 ± 8.44	12.60 ± 5.47	12.58 ± 5.46	LW + CT	8	②④	Unreported
Xu Yingbo 2021^[31]^	50/50	28/22	30/20	39.21 ± 2.74	38.83 ± 2.96	–	–	JS + CT	3	③	Unreported
Ma Jiaping 2020^[32]^	41/41	24/17	20/21	64.10 ± 2.30	63.20 ± 2.40	–	–	ZB + CT	4	①②③	Unreported
Cao Zhanhua 2013^[33]^	36/36	22/14	20/16	52.10 ± 3.70	51.70 ± 3.20	6.10 ± 2.40	6.30 ± 2.60	LW + CT	8	②④	Report
Ge Lijun 2013^[34]^	35/30	22/13	16/14	57.00 ± 4.20	58.00 ± 5.10			LW + CT	8	①②④	Unreported
Yuan Feng 2016^[35]^	36/35	18/18	18/17	54.40 ± 5.50	54.2 ± 5.8	3.70 ± 0.80	3.60 ± 0.80	LW + CT	8	①③④	Unreported
Shi Aimei 2022^[36]^	98/98	73/25	68/30	62.78 ± 8.51	58.78 ± 7.32	8.18 ± 3.13	7.63 ± 2.25	ZB + CT	4	③④	Report
Liu Mengzhen 2013^[37]^	28/28	15/13	18/10	53.60 ± 5.10	54.50 ± 6.20	9.40 ± 1.80	9.90 ± 1.90	ZB + CT	8	①④	Unreported
Liang Xuefang 2012^[38]^	27/27	17/10	18/9	57.80 ± 15.30	58.20 ± 14.80	11.20 ± 8.60	10.90 ± 8.50	JG + CT	4	②③	Unreported
Li Fuping 2011^[39]^	15/10	9/6	6/4	53.10 ± 10.20	59.20 ± 12.60	9.33 ± 3.79	8.25 ± 5.16	LW + CT	12	①③④	Unreported
Wang Yijun 2013^[40]^	33/30	18/15	16/14	60.20 ± 5.10	59.90 ± 4.80	12.50 ± 1.20	l1.90 ± 0.690	LW + CT	4	①	Report
Wang Jiuxiang 2015^[41]^	50/50	26/24	22/28	65.24 ± 8.63	64.96 ± 7.65	11.56 ± 1.22	10.45 ± 1.44	LW + CT	8	①③	Report
Wen Pingfan 2011^[42]^	20/20	9/11	10/10	52.50 ± 11.50	50.10 ± 10.70	12.50 ± 4.00	11.00 ± 5.00	LW + CT	4	②③	Unreported
Yi Yuanyue 2016^[43]^	34/34	16/18	17/17	53.00 ± 1.40	52.00 ± 1.40	–	–	LW + CT	–	②③	Unreported
Chen Bing 2009^[44]^	34/34	18/16	20/14	46.83 ± 13.25	44.29 ± 14.12	–	–	LW + CT	–	①③④	Unreported

### 3.3. Literature quality evaluation

**Figure 2.**
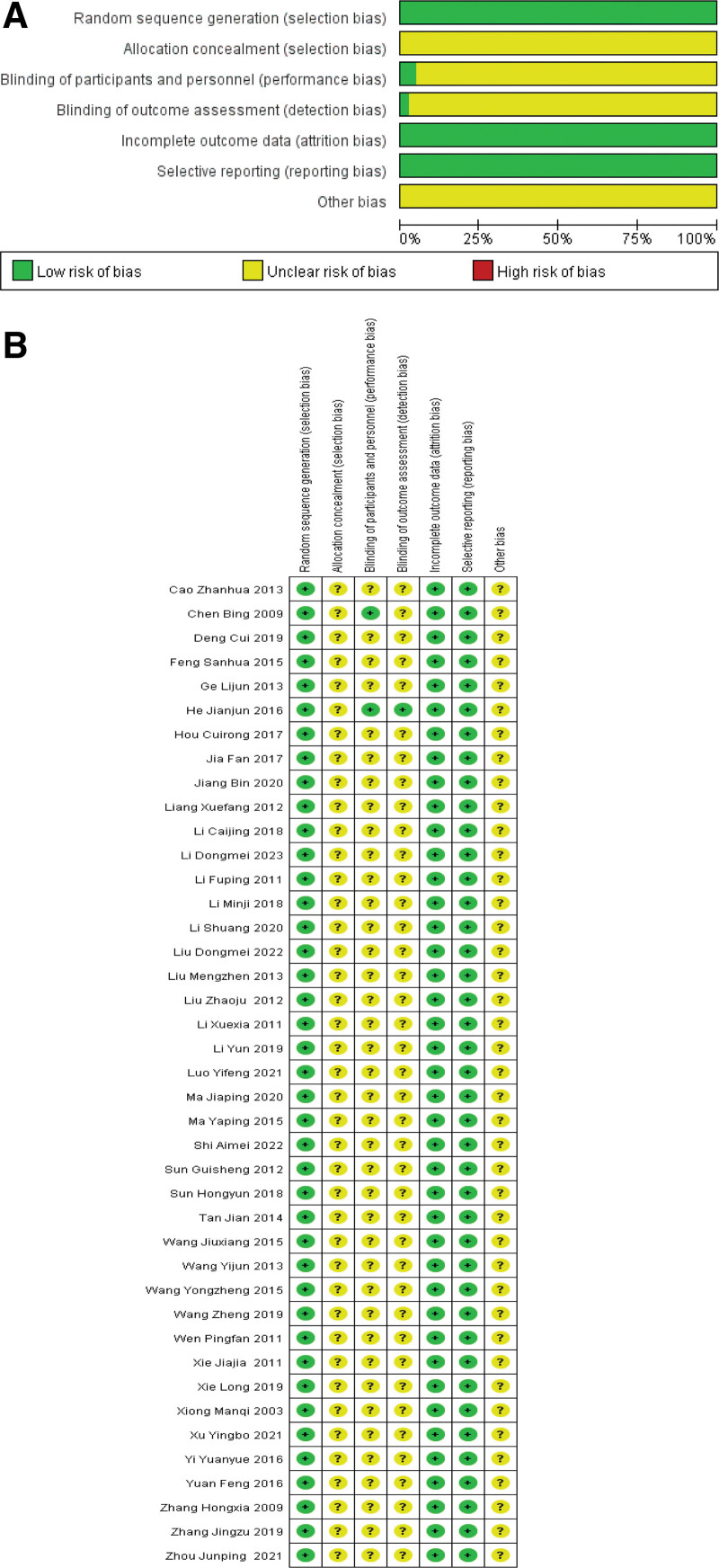
Percentages of items of included articles that produced risks of bias.

All the 41 literatures included were Chinese RCTS, and the baseline data of the experimental group and the control group were comparable. Seventeen^[4,8,11,16,17,20,22–24,26,28–31,35–37]^ items were randomly allocated by random number table method, 1^[41]^ item was randomly allocated by lottery method, 1^[40]^ item was randomly allocated by coin toss method, and the rest were explicitly proposed for random allocation and rated as “low risk.” None of the 41 studies described distribution hiding and rated as “unclear risk.” 1 study used single-blind personnel allocation and rated as “low risk”; 1 study used double-blind personnel allocation and rated as “low risk”; 39 cases did not report the use of blind method, rated as “unclear risk.” The outcome data of all literatures were complete and no selective reporting bias was found, which was rated as low risk. Other sources of bias are unclear and all rated as uncertain risk. The bias risk assessment is shown in Figure 2.

### 3.4. Network meta-analysis

#### 3.4.1. 24-hour urinary protein.

##### 3.4.1.1. Network diagram

**Figure 3.**
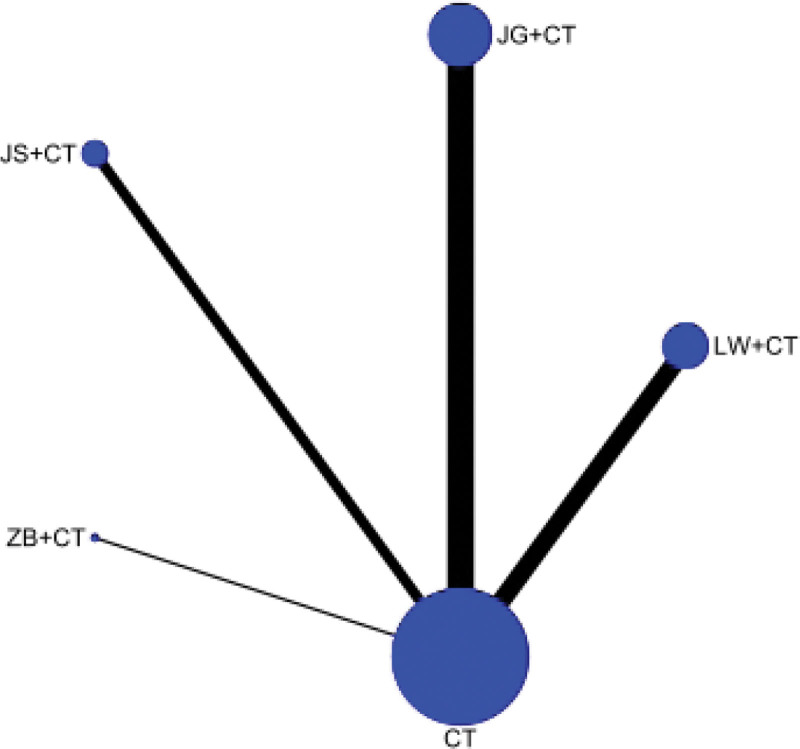
Evidence network of 24-hour urinary protein (24hUTP).

24-hour urinary protein was reported in 22 RCTS, involving 4 kinds of Dihuang pill prescriptions. The network relationship was centered on Western medicine routine, and the dot size represented the sample size of intervention measures. Therefore, Liuwei Dihuang Pills + Western medicine routine and Western medicine routine had the largest number of literatures and the largest sample size. There is no closed loop, so no inconsistency check is required. As shown in Figure 3.

##### 3.4.1.2. Network meta-analysis

**Figure 4.**
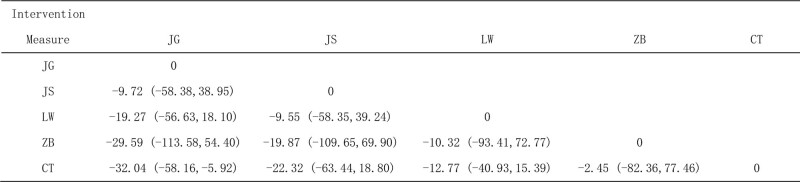
Network Meta-analysis of 24-hour urinary protein (24hUTP).

A network meta-analysis of the included studies produced 10 pairwise comparisons, and the results showed that LW + CT, JG + CT, JS + CT and ZB + CT were all superior to CT, while the other comparison differences were not statistically significant, as shown in Figure 4.

##### 3.4.1.3. SUCRA probability sort

**Figure 5.**
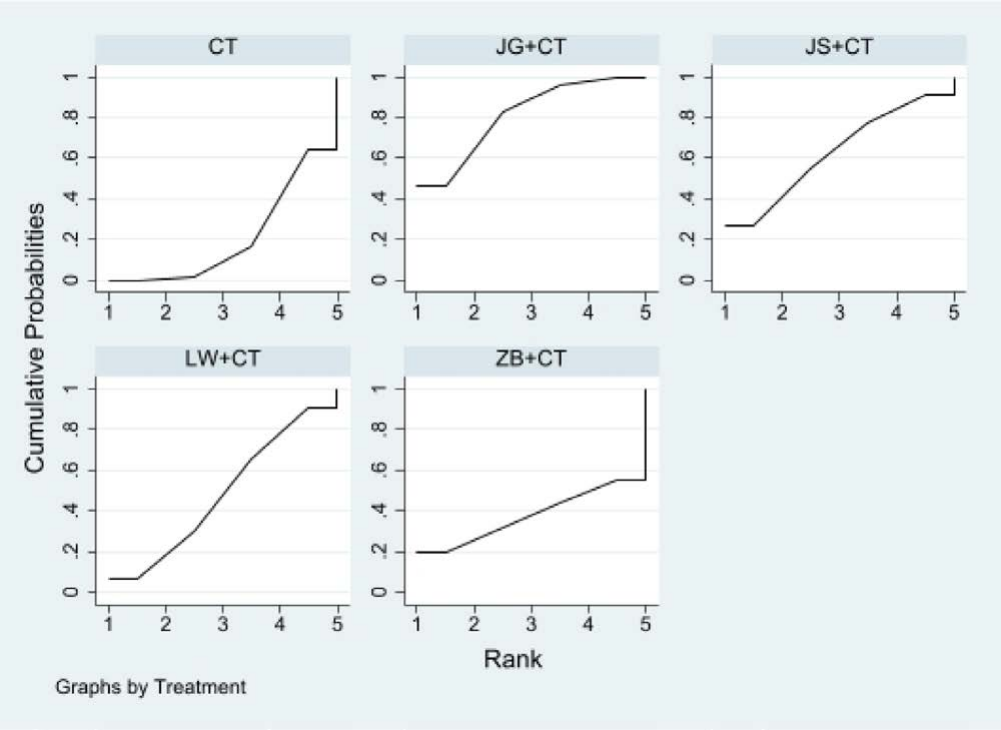
Ranking probability of 24-hour urinary protein (24hUTP).

Proprietary Chinese medicine in combination with conventional western medicine reduces 24 hutp levels, Jingui Shenqi Pills/decoction + conventional western medicine may be the most effective interventions, SUCRA The probability ranking is Jingui Shenqi Pillss/decoction + Western medicine routine > Jisheng Shenqi Pills/decoction + Western medicine routine > Liuwei Dihuang Pills/decoction + Western medicine routine > Zhibai Dihuang Pills/decoction + Western medicine routine, as shown in Figure 5.

#### 3.4.2. Urinary albumin excretion rates (UAER).

##### 3.4.2.1. Network diagram

**Figure 6.**
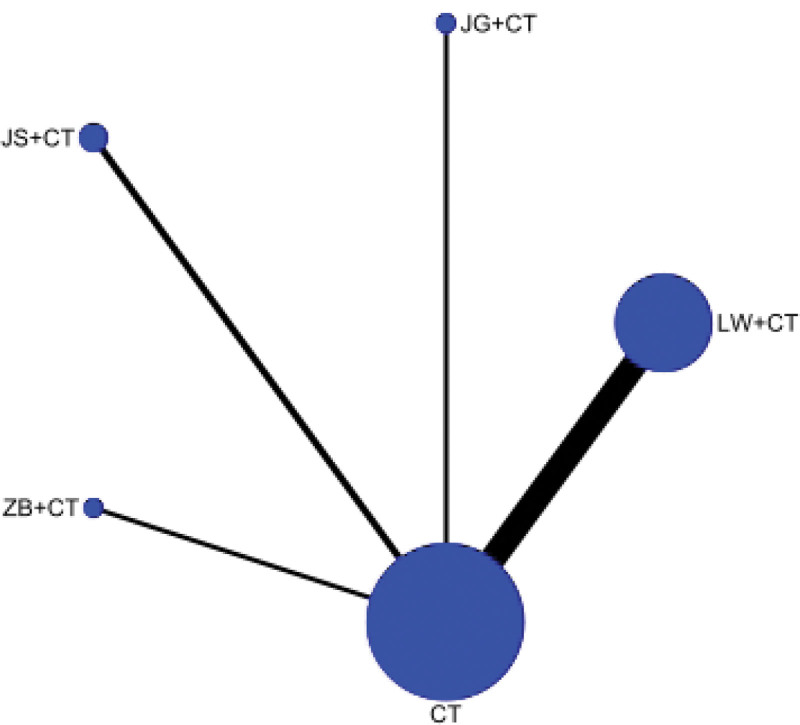
Evidence network of urinary albumin excretion rates (UAER).

22 RCTS reported UAER, involving 4 kinds of “Dihuang Pills/decoction.” The network relationship was centered on western medicine routine, and the dot size represents the sample size of the intervention, which shows that the literature volume of Liuwei Dihuang Pills + Western medicine routine and Western medicine routine was the largest, and the largest sample size. There is no closed ring, so inconsistency testing is unnecessary. As shown in Figure 6.

##### 3.4.2.2. Network meta-analysis

**Figure 7.**
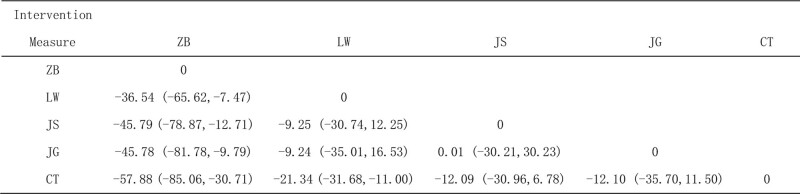
Network meta-analysis of urinary albumin excretion rates (UAER).

Through the network Meta-analysis of the included studies, a total of 10 pairwise comparisons were generated, and the results showed that: Zhibai Dihuang Pills + Western medicine routine, Liuwei Dihuang Pills + Western medicine routine, Jisheng Shenqi Pills + Western medicine routine, Jingui Shenqi Pills + Western medicine routine were better than Western medicine routine, and the other comparison differences were not statistically significant, as shown in Figure 7.

##### 3.4.2.3. SUCRA probability ranking

**Figure 8.**
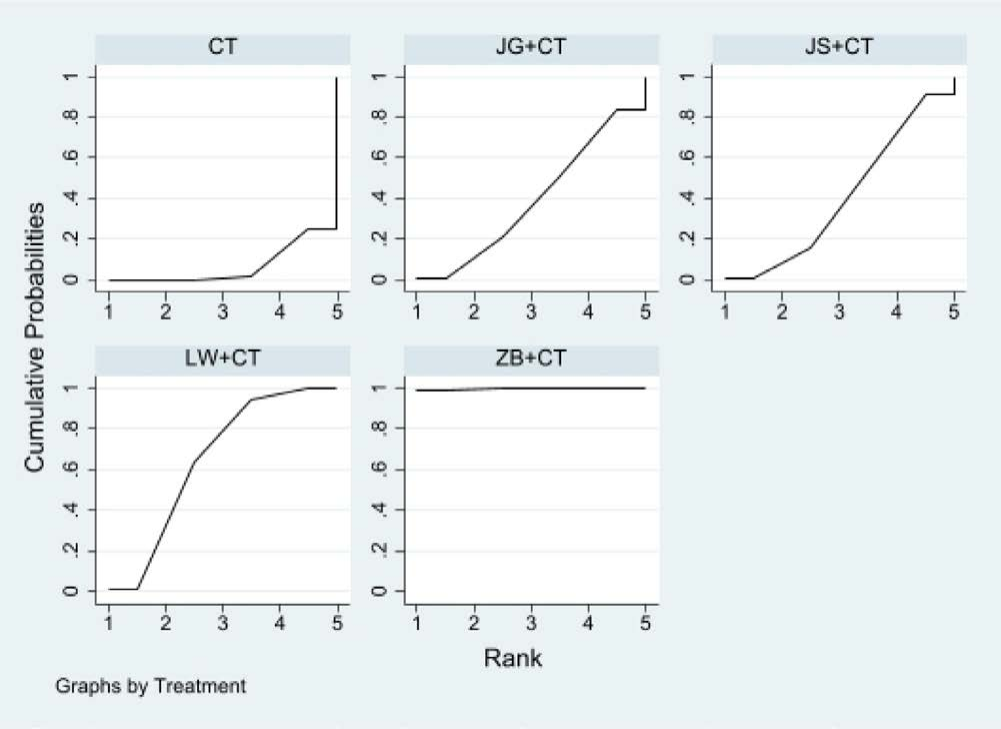
Ranking probability of urinary albumin excretion rates (UAER).

Traditional Chinese medicine and western medicine routine program to reduce the UAER level, Zhibai Dihuang Pills + Western medicine routine may be the most effective intervention measures, SUCRA probability order to know Zhibai Dihuang Pills + Western medicine routine > Liuwei Dihuang Pills + Western medicine routine > Jisheng Shenqi Pills + Western medicine routine > Jingui Shenqi Pills + Western medicine routine > Western medicine routine, as shown in Figure 8.

#### 3.4.3. Serum creatinine (Scr).

##### 3.4.3.1. Network meta-analysis

**Figure 9.**
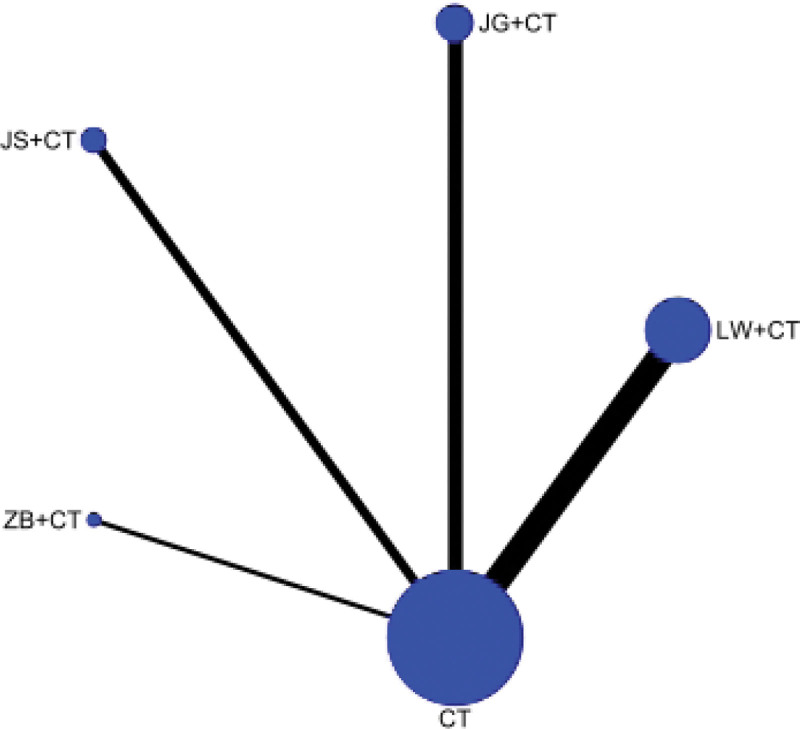
Evidence network of serum creatinine (Scr).

23 RCTS reported Scr, involving 4 kinds of “Dihuang pill prescriptions.” The network relationship was centered on western medicine routine, and the dot size represents the sample size of the intervention, which shows that the Liuwei Dihuang pills + Western medicine routine and Western medicine routine had the most literature volume and the largest sample size. There is no closed ring, so inconsistency testing is unnecessary. As shown in Figure 9.

##### 3.4.3.2. Network meta-analysis

**Figure 10.**
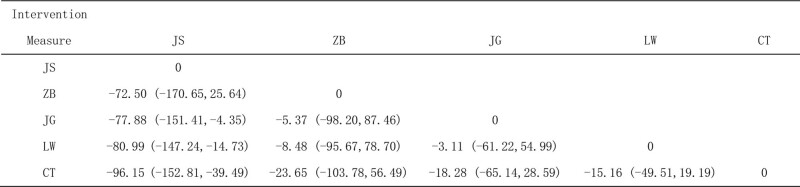
Network Meta-analysis of serum creatinine (Scr).

Through the network Meta-analysis of the included studies, a total of 10 pairwise comparisons were generated, and the results showed that: Zhibai Dihuang pills + Western medicine routine, Liuwei Dihuang pills + Western medicine routine, Jisheng Shenqi pills + Western medicine routine, Jingui Shenqi pills + Western medicine routine were better than Western medicine routine, and the other comparison differences were not statistically significant, as shown in Figure 10.

##### 3.4.3.3. SUCRA probability ranking

In terms of UAER level reduction between Chinese patent medicine and Western medicine and routine regimen, Jisheng kidney qi pill/ soup + Western medicine routine may be the most effective.

**Figure 11.**
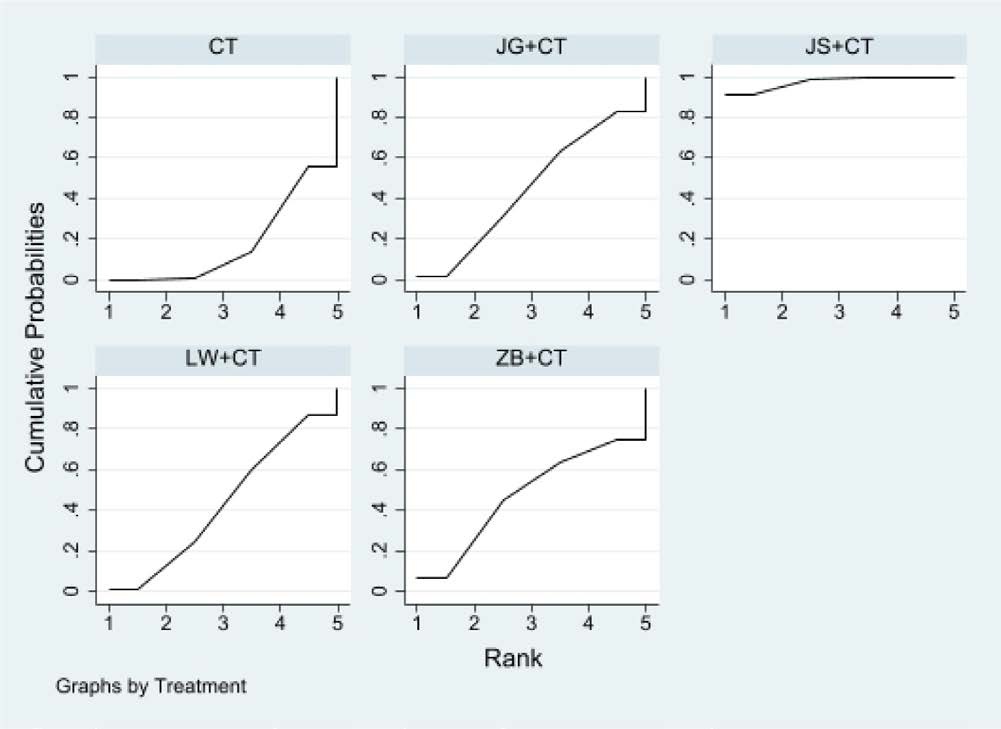
Ranking probability of serum creatinine (Scr).

Interventions, SUCRA probability ranking: Jisheng Shenqi pills/decoction + Western medicine routine > ZhiDihuang pills/ decoction + Western medicine routine > Jingui Shenqi pills/ decoction + Western medicine routine > Liuwei Dihuang pilsl/ decoction + Western medicine routine > Western medicine routine, as shown in Figure 11.

#### 3.4.4. Fasting blood glucose (FBG).

##### 3.4.4.1. Evidence network

**Figure 12.**
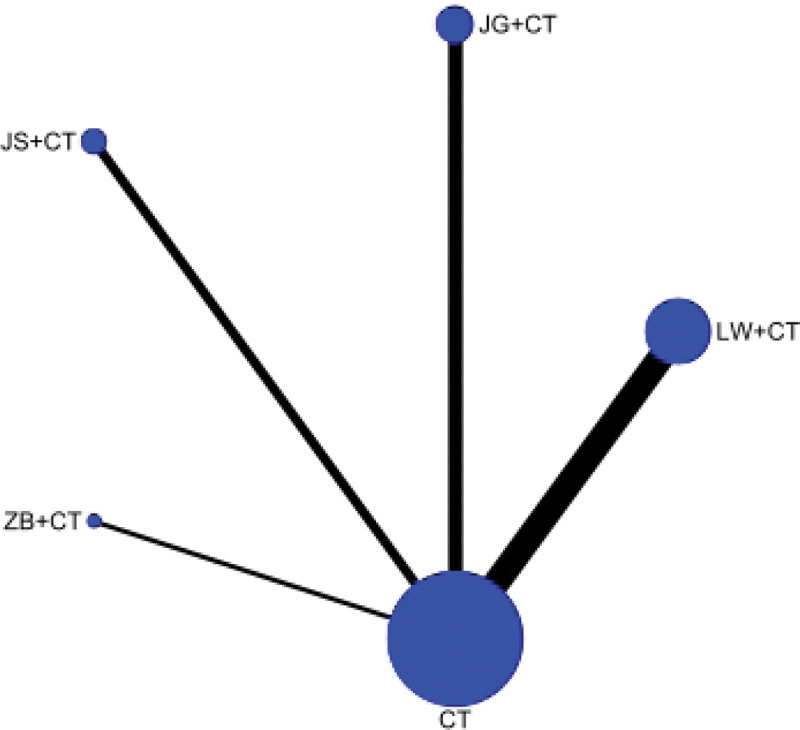
Evidence network of fasting blood glucose (FBG).

26 RCTS reported FBG, involving 4 kinds of “Dihuang pill prescriptions.” The network relationship was centered on western medicine routine, and the dot size represents the sample size of the intervention, which shows that the routine of Liuwei Dihuang pills + western medicine and western medicine had the most literature and the largest sample size. There is no closed ring, so inconsistency testing is unnecessary. See Figure 12.

##### 3.4.4.2. Network meta-analysis

**Figure 13.**
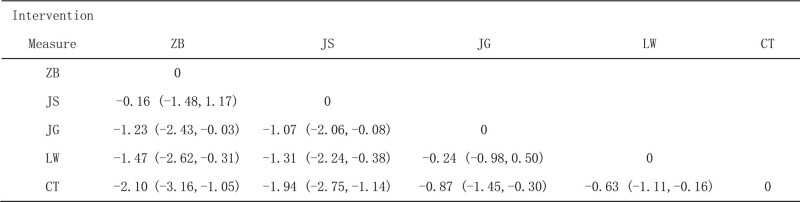
Network meta-analysis of fasting blood glucose (FBG).

Through network Meta analysis into the study, a total of 10 pairwise comparison, the results show: Zhibai Dihuang pills/decoction + Western medicine routine is better than water capsule, JiSheng Shenqi pills/decoction + Western medicine routine is superior to Western medicine routine, Jingui Shenqi pills/ decoction + Western medicine routine is better than Western medicine routine, Liuwei Dihuang pills/ decoction + Western medicine routine were better than Western medicine routine, the rest of the differences are no statistical significance, see Figure 13.

##### 3.4.4.3. SUCRA probability ranking

**Figure 14.**
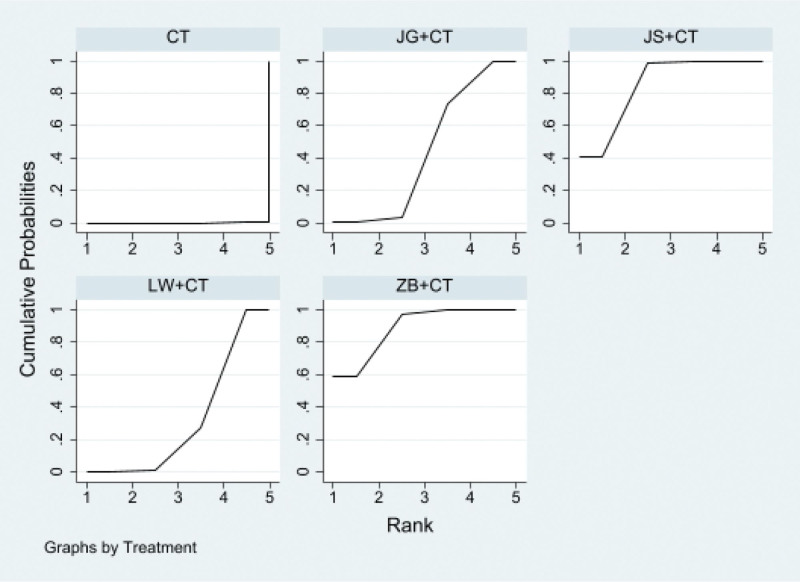
Ranking probability of fasting blood glucose (FBG).

“Dihuang pill prescriptions” joint western medicine conventional scheme to reduce the level of FBG, Zhibai Dihuang pills/ decoction + Western medicine routine may be the most effective interventions, SUCRA probability sorting for Zhibai Dihuang pills/ decoction + western medicine routine > JiSheng Shenqi pills/decoction > western medicine routine > Jingui Shenqi pills/ decoction + western medicine regular > Liuwei Dihuang pills/ decoction + western medicine routine > six western medicine routine, see Figure 14.

### 3.5. Safety

Of the 41 RCTS, 9 RCTS reported adverse reactions, including none in the study group, 3 reported dizziness and headache, 2 reported flushing, 2 reported rash, 1 reported palpitations, 1 reported digestive tract symptoms. Descriptive analysis was made because to the little literature reporting adverse reactions.

## 4. Discussion

The causes of the pathogenesis of diabetic kidney disease are extremely complex, and the specific mechanism of the pathogenesis has not been accurately and clearly stated. In the current research discussion, most people believe that the development mechanism of diabetic kidney disease, hemodynamic abnormalities, glucose and lipid metabolism disorders and inflammatory response all play a large role. Therefore, in the process of diabetic nephropathy, hyperglycemia, hypertension, abnormal lipid metabolism, overweight, obesity, and family history are all common risk factors. In the treatment of diabetic nephropathy, although the simple conventional treatment of western medicine has a good effect, it still improves the change of symptoms and the curative effect of patients. However, the use of TCM still needs to be applied clinically according to the patient constitution. With the modernization of traditional Chinese medicine, more and more traditional Chinese medicine preparations have been used in clinical, traditional Chinese medicine and western medicine has also achieved good effect, has been widely used in the clinical treatment of diabetic nephropathy.

## 5. Conclusion

“Dihuang pill prescription” is a commonly used prescription for the treatment of thirst elimination and kidney disease in ancient China. It has a history of nearly 2000 years, and it is still widely used in the clinical diagnosis and treatment of diabetic nephropathy. And after a large number of clinical studies, it shows that the combination of traditional Chinese medicine and western medicine plays an important role in the prevention and treatment of diabetic nephropathy, which can delay the progression of the disease and reduce the possibility of deterioration of the disease into end-stage kidney disease. The above studies still have some limitations, so more high-quality, multicenter, large sample and late follow-up RCT are needed to supplement and improve.

This study involved 4 types of “Dihuang pill prescriptions,” including Liuwei Dihuang pill/soup, Jinkui Renqi pill soup, Jisheng Renqi pill/soup, and Zhibaidihuang pill/soup. The comprehensive results of network Meta analysis suggest that the efficacy of oral Chinese medicine combined with conventional Western medicine for diabetic nephropathy is often better than that of conventional western medicine treatment alone, with no serious adverse effects. Among them, Jinkui Renqi pill/ soup + Western medicine routine, Jisheng kidney qi pill/ soup + Western medicine routine can significantly reduce UAER; Zhibaidihuang pill/ soup + Western medicine routine, Liuwei Dihuang pill/ soup + Western medicine routine has more advantages in reducing 24 hours UTP; in reducing Scr, Jisheng Renenqi pill/ soup + Western medicine routine, Zhibaidihuang pill/ soup + Western medicine routine has more advantages; in reducing fasting blood glucose, Zhibaidihuang pill/ soup + Western medicine routine, Jisheng qi pill/ soup + Western medicine routine has more advantages. Therefore, in the clinical treatment of diabetic nephropathy, intervention should also be conducted according to the course of diabetic nephropathy. If proteinuria, recommend Liuwei Dihuang pill/ soup and Western medicine routine, renal qi pill/ soup + Western medicine routine; if Scr increased, recommend JiSheng Renqi pill/ soup + Western medicine routine, bai Dihuang pill/ soup + Western medicine routine. The above interventions are for clinical reference only, but they still need to combine the clinical manifestations and normal constitution of patients.

This paper also has some defects, mainly in the following 3 aspects: The quality of the included literature still needs to be improved, among which only 2 articles mentioned the blind method, all studies did not report whether the allocation concealment, there is a large risk bias in the results. The western medicine routines involved in this paper are different from the same, and the dosage form, dosage are different, the course of treatment is different, will also have a certain impact on the results of this paper. Among the 41 articles included in the analysis, there was a lack of follow-up of patients, such as whether to prolong the progression of diabetic nephropathy and the incidence of progression to end-stage renal disease.

## Author contributions

**Conceptualization:** Minghao Lin.

**Formal analysis:** Minghao Lin.

**Methodology:** Minghao Lin.

**Project administration:** Hui Zhang.

**Resources:** Hui Zhang.

**Software:** Hui Zhang, Shilin Liu, Andong Li.

**Supervision:** Zheng Nan.

**Writing – original draft:** Minghao Lin.
